# PUFA diets alter the microRNA expression profiles in an inflammation rat model

**DOI:** 10.3892/mmr.2015.3318

**Published:** 2015-02-09

**Authors:** ZHENG ZHENG, YINLIN GE, JINYU ZHANG, MEILAN XUE, QUAN LI, DONGLIANG LIN, WENHUI MA

**Affiliations:** 1 Department of Biochemistry and Molecular Biology, Medical College, Qingdao University, P.R. China; 2 Department of Pathology, Affiliated Hospital of Qingdao University, Qingdao, Shandong 266021, P.R. China

**Keywords:** microRNA, polyunsaturated fatty acids, immune homeostasis, microRNA target gene prediction, functional enrichment analysis

## Abstract

Omega-3 and -6 polyunsaturated fatty acids (PUFAs) can directly or indirectly regulate immune homeostasis via inflammatory pathways, and components of these pathways are crucial targets of microRNAs (miRNAs). However, no study has examined the changes in the miRNA transcriptome during PUFA-regulated inflammatory processes. Here, we established PUFA diet-induced autoimmune-prone (AP) and autoimmune-averse (AA) rat models, and studied their physical characteristics and immune status. Additionally, miRNA expression patterns in the rat models were compared using microarray assays and bioinformatic methods. A total of 54 miRNAs were differentially expressed in common between the AP and the AA rats, and the changes in rno-miR-19b-3p, -146b-5p and -183-5p expression were validated using stem-loop reverse transcription-quantitative polymerase chain reaction. To better understand the mechanisms underlying PUFA-regulated miRNA changes during inflammation, computational algorithms and biological databases were used to identify the target genes of the three validated miRNAs. Furthermore, Gene Ontology (GO) term annotation and KEGG pathway analyses of the miRNA targets further allowed to explore the potential implication of the miRNAs in inflammatory pathways. The predicted PUFA-regulated inflammatory pathways included the Toll-like receptor (TLR), T cell receptor (TCR), NOD-like receptor (NLR), RIG-I-like receptor (RLR), mitogen-activated protein kinase (MAPK) and the transforming growth factor-β (TGF-β) pathway. This study is the first report, to the best of our knowledge, on *in vivo* comparative profiling of miRNA transcriptomes in PUFA diet-induced inflammatory rat models using a microarray approach. The results provide a useful resource for future investigation of the role of PUFA-regulated miRNAs in immune homeostasis.

## Introduction

microRNAs (miRNAs) are small, non-coding, endogenous RNAs that regulate cellular gene expression during or after transcription by base pairing with their target transcripts, thereby suppressing the initiation of translation, inhibiting elongation, and inducing deadenylation, which can decrease mRNA stability and increase mRNA degradation (1–4). Most miRNAs are evolutionarily conserved in related species, while it was previously shown that certain miRNAs can regulate a wide range of biological processes, as well as disease development and progression (5).

Both omega-3 and -6 polyunsaturated fatty acids (PUFAs) can affect cellular gene expression. Generally, omega-3 PUFAs compete with omega-6 ones to change the composition of fatty acids in the plasma and thereby exert their anti-inflammatory and immunomodulatory effects (6). In particular, dietary omega-3 PUFAs can suppress circulating pro-inflammatory cytokines, such as interleukin-6 (IL-6), C-reactive protein (CRP), and tumor necrosis factor-α (TNF-α), by increasing the levels of the E3 series of prostaglandins, 5 series of leukotrienes, resolvin and protectin, whereas omega-6 PUFAs exert pro-inflammatory effects by increasing the levels of E2 series of prostaglandins and of the 4 series of leukotrienes. Considering that inflammation is at the origin of numerous chronic diseases, additional dietary omega-3 PUFAs are crucial for human health (7).

Accumulating data have demonstrated that PUFAs exert regulatory effects by directly or indirectly modulating miRNA expression. During the past decade, the impacts of daily fatty acid intake on miRNA biogenesis, function and decay were intensively studied. Recent studies indicated that miRNA dysfunction caused by improper PUFA intake is significantly associated with numerous metabolic diseases, autoimmune (AI) diseases, obesity, and cancer (8,9).

Although PUFA-regulated miRNAs can post-transcriptionally regulate various developmental and physiological processes, the precise miRNA expression patterns induced by certain PUFAs and their biological functions in immune homeostasis remain elusive.

We aimed to compare the miRNA profiles of rats fed with a PUFA diet to the profiles of control rats, and to identify whether omega-3 or -6 PUFAs can change the expression patterns of specific miRNAs in certain tissues. Using bioinformatic analysis, we evaluated the effects of PUFA-supplemented diets on immune homeostasis, and further propose a simple and clear model of the complex relationship among PUFAs, miRNAs, target genes, and biological processes. This model may facilitate researchers in further exploring the immune processes controlled by the PUFA-regulated miRNAs.

## Materials and methods

### Ethics

All experiments on animals were conducted according to the National Institutes of Health Guide for Care and Use of Laboratory Animals (publication 85–23, revised 1985). The protocols used in this study were approved by the Review Committee for the Use of Human or Animal Subjects of the Medical College, Qingdao University (permit no. AS-2012019). All surgical procedures were performed under sodium pentobarbital-induced anesthesia, and efforts were made to minimize suffering of the animals.

### Animals and diets

A total of 30 three-week-old Wistar rats were obtained from the Shanghai Laboratory Animal Center (SLAC; Chinese Academy of Sciences, Shanghai, China) and were allowed to acclimatize to the animal facility conditions (constant humidity 50%, 20±2°C, and 12 h dark/light cycle) for 1 week before the experiments were performed. All animals were provided with water and AIN-93G rat chow (SLAC) mixed with a blend of lard and corn oil; this diet mimics common human diets, which include high saturated fatty acids, single unsaturated fatty acids, and omega-6 PUFAs, but low omega-3 PUFAs (7). We randomly divided the rats into three groups of equal size: the omega-3 PUFA diet-induced autoimmune-averse (AA), the omega-6 PUFA diet-induced autoimmune-prone (AP) and the control (Ct) rat group. The rats from the omega-3 and omega-6 PUFA-treated groups were fed with a mixture of the omega-3 PUFAs (10 *μ*l/100 g/day) eicosapentaenoic acid (EPA) and docosahexaenoic acid (DHA) at a ratio of 1.5:1, and with the omega-6 PUFA linoleic acid, respectively (all from Sigma-Aldrich, St. Louis, MO, USA), by gentle intragastric administration every two days; the Ct rats were fed with a 0.9% saline solution (10 *μ*l/100 g/day) instead. The animals were fed for 16 weeks, and body weight and naso-anal length were recorded weekly.

### Tissue collection and RNA extraction

The animals were humanely sacrificed by an overdose of sodium pentobarbital (Solarbio Science & Technology Co., Ltd., Beijing, China) at week 16. Peripheral blood mononuclear cells (PBMCs) were collected from blood (anticoagulated with EDTA-Na2) using Ficoll-Paque Premium 1.084 solution (GE Healthcare, Piscataway, NJ, USA). Omental fat, visceral fat, liver, spleen, brain, hypophysis and peripheral blood serum were rapidly separated from each animal, weighed, immediately frozen in liquid nitrogen, and stored at −80°C. Frozen tissues and PBMCs were homogenized in RNAiso for Small RNA (Takara, Dalian, China), and the miRNAs were isolated from white adipose tissue, hepatocytes, PBMCs, and peripheral blood serum according to the manufacturer’s protocol. The quality of the extracted RNAs was verified using a NanoDrop ND-1000 spectrophotometer (Thermo Fisher Scientific, Waltham, MA, USA) and agarose gel analysis.

### Hematoxylin and eosin (H&E) staining

The same region of the omental fat pad was excised from the rats, fixed in paraformaldehyde, embedded in paraffin, cut into 5-*μ*m sections, and stained using the H&E staining kit (Beyotime Institute of Biotechnology, Jiangsu, China) according to the manufacturer’s instructions. The surface areas of the adipocytes were measured on images acquired from examination under an inverted microscope at x100 magnification (ECLIPSE TS100; Nikon, Tokyo, Japan) using the lasso tool in Photoshop CS4 (Adobe Systems, Inc., San José, CA, USA). The data were compared with a one-way analysis of variance (ANOVA) using the GraphPad Prism 5 software (GraphPad Software, San Diego, CA, USA).

### Flow cytometry

To perform three-color flow cytometry analysis, fresh PBMCs were collected, washed and resuspended in phosphate-buffered saline containing 1% newborn bovine serum (Thermo Fisher Scientific). PBMCs were stained with anti-rat CD4-fluorescein isothiocyanate (FITC), CD3-phycoerythrin (PE) and CD8a-PE-Cy7 to perform CD4^+^/CD8^+^ T-lymphocyte measurements. To assess the population of regulatory T cells (Tregs) in the peripheral blood, PBMCs were first stained with anti-rat CD4-FITC and CD25-peridinin chlorophyll (PerCP)-eFluor 710, and then stabilized and permeabilized using the FoxP3 staining buffer set to allow anti-rat Foxp3-PE to stain intracellularly. Anti-rat IgG2a-PE and IgG1-PerCP-eFluor 710 were used as the isotype controls. All reagents for the flow cytometry analysis were purchased from eBioscience (San Diego, CA, USA). Fluorescence was measured on an FC 500 flow cytometer, and the data were collected and analyzed using the CXP 2.1 software (both from Beckman Coulter, Brea, CA, USA). All procedures were performed as per the manufacturers’ instructions. One-way ANOVA was performed on log_2_-transformed raw data from flow cytometry using the GraphPad Prism 5 software.

### Enzyme-linked immunosorbent assay (ELISA) assay

The stored serum samples of the rats were used to determine the levels of inflammatory markers. High-sensitivity sandwich ELISA tests for IL-6, CRP and TNF-α were conducted in duplicate using the corresponding ELISA kits (Abcam, Cambridge, UK) according to the manufacturers’ instructions. The relative concentrations of the markers were determined by analyzing raw optical density (OD) data, measured on a Denley Dragon Wellscan MK 3 spectrophotometer (Thermo Fisher Scientific, Vantaa, Finland). Data analysis was performed using the curve-fitting software ReaderFit (Hitashi Solutions America, Ltd., San Bruno, CA, USA) with a 4-parameter logistic regression.

### Microarray assay and significance analysis

Three rats were randomly selected from each group, and global miRNA expression in their peripheral blood serum was analyzed using the miRCURY LNA™ Array 13.0 (Exiqon, Vedbæk, Denmark). Microarray scanning was performed with an Axon GenePix 4000B Microarray scanner (Molecular Devices, Sunnyvale, CA, USA). The raw data were normalized after miRNA microarray processing with the GenePix Pro 6.0 software (Molecular Devices), using the per-chip median normalization method. The Significance Analysis of Microarrays (SAM) software (10) was used to define the up- and downregulated miRNAs (fold-change >2, P<0.05, SAM δ-value >4.5) (11). Hierarchical clustering was performed using Cluster 3.0 (12) and Java TreeView (version 1.1.6r2) (13); the original microarray data were transformed to log_2_ values and visualized as a heatmap. Pearson’s correlation (uncentered correlation) and the centroid linkage rule were used in unsupervised clustering to group the similarly regulated miRNAs. Permutation tests were implemented in BRB-ArrayTools (http://linus.nci.nih.gov/BRB-ArrayTools.html) by permuting the normalized intensity of each miRNA probe, in order to identify whether the miRNAs were differentially expressed between the groups. Permutation p-values were based on 10,000 random permutations. The raw data from the miRNA microarrays were deposited in ArrayExpress under the accession number E-MTAB-1615.

### Stem-loop reverse transcription-quantitative polymerase chain reaction (RT-qPCR)

Since stem-loop RT primers show better efficiency and specificity compared to conventional primers (14), the RT reaction was conducted using a RevertAid First Strand cDNA Synthesis kit (Thermo Fisher Scientific) and stem-loop RT primers (Guangzhou RiboBio Co., Ltd., Guangzhou, China). Fermentas^®^ RNase-free DNase I (Thermo Fisher Scientific) was used to remove genomic DNA from the RNA samples. qPCR was performed with the FastStart Universal SYBR-Green Master mix (Roche Applied Science, Indianapolis, IN, USA) on a Corbett Rotor-Gene 3000 system (Qiagen, Valencia, CA, USA). Following amplification, melting-curve analysis was performed as described by the manufacturer. Each RNA sample was analyzed in triplicate, and all measurements contained a negative control with no cDNA template and an endogenous control with an rno-U6 small nuclear RNA (snRNA). The relative expression levels of the miRNAs were calculated with the comparative cycle threshold (Ct) method using the REST 2009 software (Qiagen) (15).

### Identifying and visualizing immune-related miRNAs

To accurately predict the miRNA targets, the list of miRNAs was imported into the miRWalk database, which uses a combination of algorithms for prediction (16,17). Then, we identified the target genes of these miRNAs, and the regulatory network of miRNA-mRNA interactions was visualized using Cytoscape (18). The official gene symbols of the target genes were retrieved, and enrichment of these genes for functional categories was performed with DAVID (19), an online service tool that uses the Kyoto Encyclopedia of Genes and Genomes (KEGG) pathways and Gene Ontology (GO) terms for functional annotation. We thus identified miRNA-targeted genes (MTGs) and the miRNA-targeted cellular pathways (MTPs) in which these genes are involved. The predicted and complex relationships between the tissues, miRNAs, MTGs and MTPs, were summarized in a graphic representation obtained using the Circos program (20).

### Statistical analysis

All values in the graphs are given as means ± standard error of the mean. To assess statistical significance, one-way ANOVA followed by Dunnett’s multiple comparison tests was performed using the GraphPad Prism 5 software. The microarray data were analyzed using SAM and Cluster 3.0. The RT-qPCR data were assessed using REST 2009. The enrichment of pathways and GO terms was performed using the online DAVID tool suite. The values obtained in the assays were considered significantly different at P<0.05.

## Results

### PUFA diets affect the physical characteristics of the rats

**Figure 1:**
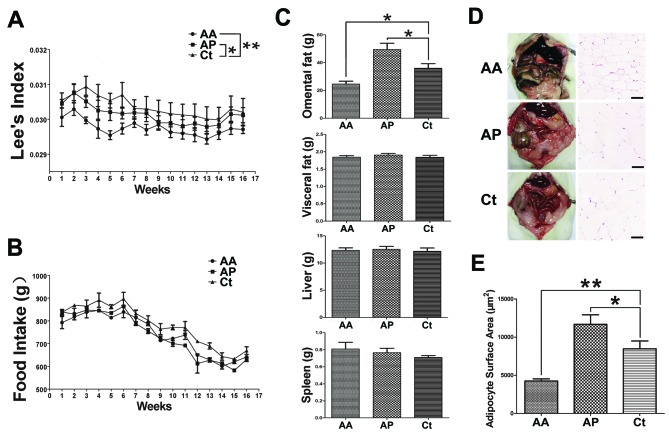
Effects of omega-3 on physical characteristics of rats. (A) Changes in Lee’s index (n=10). (B) The weekly food consumption survey reveals equal food intake for all groups (n=10). (C) Weight of omental fat, visceral fat, liver, and spleen tissue (n=10). (D) Hematoxylin and eosin staining histology. Scale bar, 50 *μ*m. (E) Adipocyte surface area (n=50). All data are expressed as means ± standard error of the mean. ^*^P<0.05 and ^**^P<0.01. AA, autoimmune-averse; AP, autimmune-prone; Ct, control.

Dynamic monitoring of the Lee’s index, which is equal to body weight (g^0.33^)/naso-anal length (mm) (21), and of the weekly total food intake, indicated that the degree of obesity is significantly lower in the AA rats compared to the Ct rats, although the food consumption in the two groups was similar (Fig. 1A and B). In comparison to the Ct rats, the AA rats exhibited a marked reduction in deposition of omental fat (Fig. 1C and D), and histochemical staining revealed significantly smaller adipocytes in the omental fat (Fig. 1D and E). Opposite trends were observed in the AP rats (Fig. 1A–E).

### PUFA diets modulate the inflammation status in vivo

**Figure 2:**
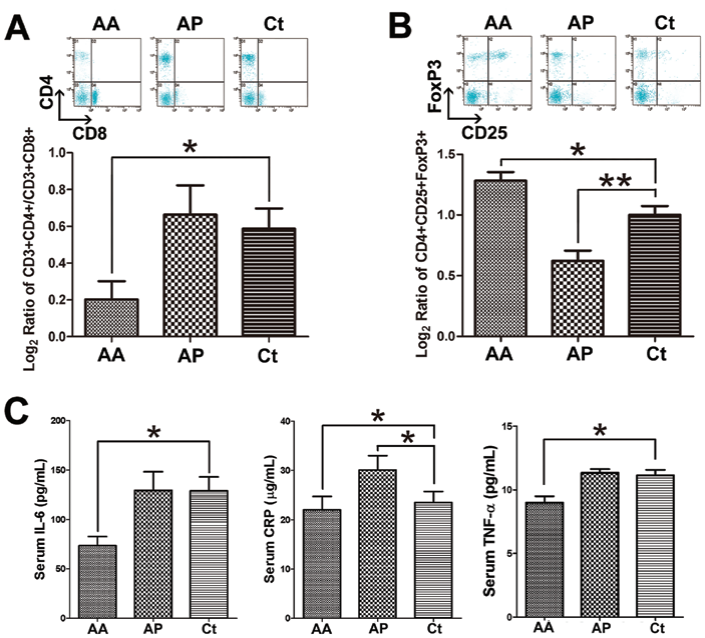
Effects of the omega-3 diet on the attenuation of immune hyperfunction in rats. (A) Representative flow cytometry plots (upper); log_2_ ratio of CD3^+^CD4^+^ T cells to CD3^+^CD8^+^ T cells (lower; n=5). (B) Representative flow cytometry plots (upper); log_2_ ratio of CD4^+^CD25^+^FoxP3^+^ regulatory T cells (lower; n=5). (C) Concentrations of serum interleukin-6 (IL-6), C-reactive protein (CRP), and tumor necrosis factor-α (TNF-α) in polyunsaturated fatty acid diet-induced rats (n=5). All data are expressed as means ± standard error of the mean. ^*^P<0.05 and ^**^P<0.01. AA, autoimmune-averse; AP, autoimmune-prone; Ct, control.

To minimize collateral damage during immune response, a complex network of interacting regulatory peripheral mechanisms has coevolved to prevent and overcome immune-mediated diseases. These regulatory systems include mechanisms intrinsic to the differentiation of suppressor T cells, such as CD8^+^ T cells and Tregs (22,23). To investigate whether PUFA diets can inhibit the inflammatory response via suppressor T cells, we used three-color flow cytometry analysis to assess the ratio of CD8^+^/CD4^+^ T cells and the level of CD4^+^CD25^+^FoxP3^+^ Tregs in rats fed with PUFA diets. The results showed that the population of CD8^+^ T cells and the population of Tregs are significantly increased in the O3 rats, whereas the population of Tregs is decreased in the O6 rats (Fig. 2A and B). In addition, ELISA assays showed that the concentrations of IL-6, CRP and TNF-α are significantly decreased following an omega-3 PUFA diet (Fig. 2C). These results indicated that the omega-3 PUFA diet exerts an anti-inflammation effect *in vivo*, by decreasing the pro-inflammatory cytokines and increasing the ratio of suppressor T cells.

### PUFA diets alter miRNA expression patterns

**Figure 3:**
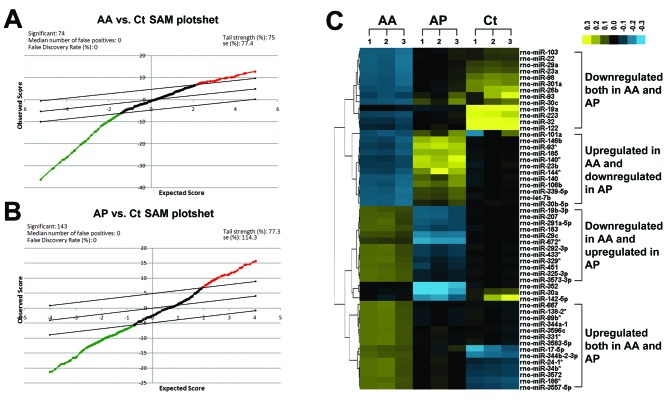
(A and B) Significance Analysis of Microarray (SAM) plot and (C) heatmap of differentially expressed microRNAs (miRNAs). The δ-value in SAM analysis was set to 5, and the false discovery rate was 0. There are (A) 74 miRNAs differentially expressed in autoimmune-averse (AA) and (B) 143 miRNAs in autoimmune-prone (AP) rats. (C) In the heatmap, significantly upregulated miRNAs are shown in yellow, and downregulated miRNAs in blue. Twenty-four miRNAs are differentially expressed in the two different polyunsaturated fatty acid (PUFA) diet treatment groups. The colored scale bar represents the log_2_-converted fold-change value. Ct, control.

Circulating miRNAs can be used to identify certain clinical conditions and can reflect environmental stress, since they are stably and consistently expressed among individuals of the same species (24). Therefore, peripheral serum was chosen as a suitable sample to study the global variation in miRNA expression patterns. Using microarray and SAM analyses, 74 and 143 miRNAs were statistically identified as differentially regulated in the AA and AP rats compared to the Ct rats (Fig. 3A and B), respectively. Of these, 54 miRNAs were identified in both the AA and the AP rat groups, and were selected for hierarchical clustering analysis (Fig. 3C). The results showed that 30 miRNAs are down- or upregulated in both PUFA-treated groups, and 24 miRNAs are oppositely regulated in the AA and AP rat groups. Based on a permutation test, the overlap between the PUFA-regulated miRNAs observed in the AA and AP rats was significant.

### Functional clustering of PUFA-regulated MTGs

Following initial identification of PUFA-regulated miRNAs by SAM and hierarchical clustering analysis, the 24 oppositely regulated miRNAs in the AA and AP groups were analyzed in order to predict their corresponding MTGs, which were further subjected to functional enrichment analysis. The miRWalk (16) database, which at present employs 5 established prediction algorithms [miRanda (25), miRDB (26), miRWalk, PITA (27), TargetScan (28)], was used to predict the MTGs. In total, 708 putative MTGs, which were positive in at least 4 of the programs, were chosen according to stringent standards (min seed length =10, P<0.01), and were pooled in MTG datasets for each miRNA. The DAVID functional annotation clustering tool was used to perform KEGG pathway and GO term enrichment analyses on these MTG datasets. The parameters and stringency of the clustering tools were set at high levels to enhance prediction accuracy. Next, the KEGG and GO MTG-related clusters that were relevant to inflammation and showed high enrichment scores were selected. According to these filtering criteria, certain miRNAs were excluded, and rno-miR-19b-3p, -29c, -146b-5p, -183-5p and -292-3p were selected as candidate miRNAs for RT-qPCR validation. The association networks relevant to the 5 candidate miRNAs and the 708 putative MTGs were also constructed and visualized using Cytoscape (data not shown).

### RT-qPCR validation of candidate miRNAs

**Figure 4:**
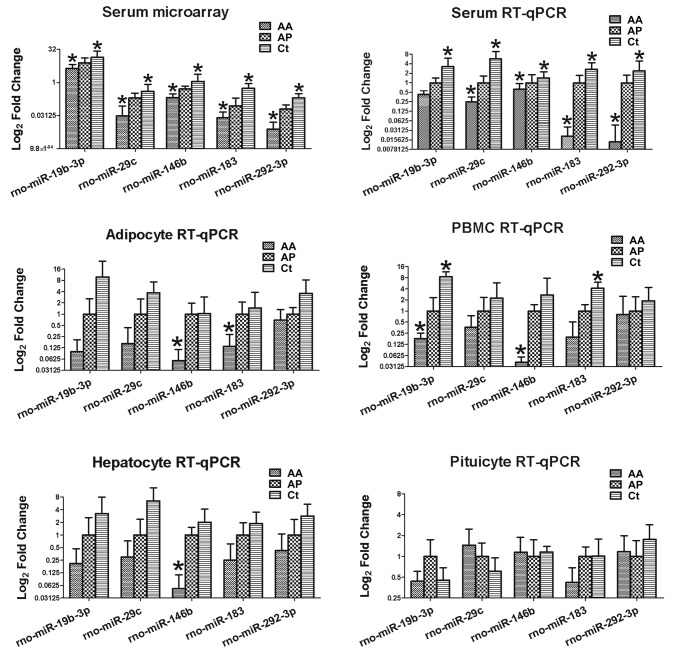
Reverse transcription-quantitative polymerase chain reaction (RT-qPCR) validation of the identified microRNAs (miRNAs). Relative expression ratio of the miRNAs in the serum as assessed by microarrays (n=3), and RT-qPCR analysis in the serum, peripheral blood mononuclear cells (PBMCs), adipocyte, hepatocyte, and pituicyte. The relative amounts of each miRNA were normalized to that of the U6 small nuclear RNA). The y-axis values were transformed to log_2_ scales, and all data are shown as means ± standard error of the mean. ^*^P<0.05 compared to the control. AA, autoimmune-averse; AP, autoimmune-prone; Ct, control.

To confirm the microarray findings and to further analyze the expression patterns of the 5 candidate miRNAs in certain tissues, the miRNA expression levels in rat serum, PBMCs, visceral adipocytes, hepatocytes and pituicytes were separately measured using RT-qPCR. The serum RT-qPCR data confirmed the microarray data (Fig. 4). However, the RT-qPCR results from other tissues did not completely agree with the microarray data (Fig. 4), most likely because of the high variability of miRNA expression in different tissues. In particular, miR-19b-3p in PBMCs showed a notable downregulation trend in the AA rats and an increase in the AP rats compared to the Ct rats, and was not differentially expressed in a significant manner in the other tissues. Moreover, in AA rats, both miR-146b-5p and -183-5p showed a significant decrease in the visceral adipose tissue, and miR-146b-5p was significantly downregulated in the PBMCs and hepatocytes. In the AP rats, miR-183-5p was upregulated in the PBMCs. These findings indicated that rno-miR-19b-3p, -146b-5p and -183-5p are regulated by the PUFA diets in the visceral fat, PBMCs, and liver tissues.

### Exploratory visual analysis of candidate immune-related miRNAs

**Figure 5:**
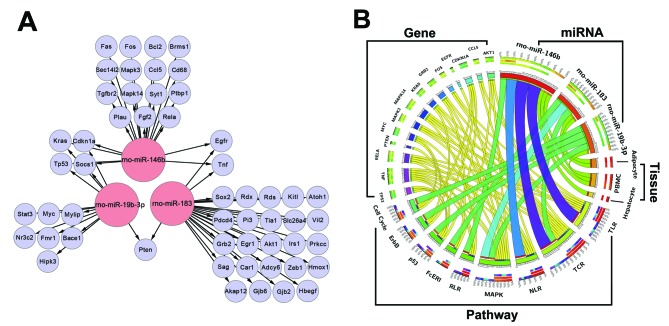
Visualization of miRNA-targeted genes (MTGs). (A) Interaction network of the microRNAs (miRNAs) and their validated MTGs. Lines indicate direct interactions between miRNAs and MTGs. Small circle nodes represent MTGs, while large circle nodes denote miRNAs. (B) Circos ideogram showing the relationships among the tissues, tissue-specific polyunsaturated fatty acid (PUFA)-regulated miRNAs, validated MTGs, and MTPs. TLR, Toll-like receptor; TCR, T cell receptor; NLR, NOD-like receptor; MAPK, mitogen-activated protein kinase; RLR, RIG-I-like receptor; FcERI, Fc ε RI; ErbB, also named epidermal growth factor receptor.

Based on the results of the RT-qPCR validation, we obtained 53 experimentally verified (16) MTGs from rno-miR-19b-3p, -146b-5p and -183-5p using the miRWalk Validated Targets tool. Fig. 5A represents the network of these miRNAs and their associated MTGs, drawn using the Cytoscape software. DAVID tools were also used to search for the KEGG pathways in which the validated MTGs are involved. The relationships among the tissues, miRNAs, MTGs and MTPs of interest are depicted in Fig. 5B in the form of an ideogram, obtained by mapping the data of all predicted associations using the Circos program. In this circular diagram, segments on the inner circle represent the miRNAs, tissues, MTGs or MTPs, and the ribbons represent the links between the segments. For instance, miR-146b-5p is involved in the TLR pathway by targeting TNF, FOS, mitogen-activated protein kinase (MAPK)14, MAPK3, RELA and CCL5 in adipocytes, hepatocytes and PBMCs. The Circos diagram clearly depicts the links among the tissues, miRNAs, genes and pathways, and this type of visualization may be helpful to researchers who wish to explore how specific miRNAs affect PUFA-regulated signaling pathways. There is currently no GO term database that can detail biological mechanisms, thus the data from GO terms that are associated with immune-related miRNAs are not shown in the Circos diagram.

## Discussion

The interaction between genes and the environment is the foundation of health and diseases. The modern nutritional environment differs from that of our ancestors, as a result of modern agriculture and agribusiness. One of the disturbing features of modern diet is the increasingly severe imbalance between omega-3 and omega-6 PUFAs, which may lead to long-chain (LC) omega-3 PUFA deficiency (7).

Dietary or therapeutic supplements of LC omega-3 PUFAs alter the composition of cell membranes and affect the expression of inflammatory genes by activating transcription factors (29). Previous studies on the cardioprotective functions of PUFAs revealed that LC omega-3 PUFAs reduce the production of pro-inflammatory leukotrienes and cytokines (30,31). Consistently, we observed a marked reduction in inflammatory markers (IL-6, CRP and TNF-α) in the AA rat group (Fig. 2C), which indicated that omega-3 PUFAs inhibit pro-inflammatory cytokines *in vivo*.

With regards to obesity-preventive mechanisms, it was previously found that the number of adipocytes in lean and obese individuals is set during childhood and adolescence (32). Consistent with this notion, we found that the omega-3 PUFA diet prevents the deposition of omental fat in juvenile female rats, while the omega-6 PUFA diet increases the accumulation of omental fat (Fig. 1A–E). Currently, obesity is accepted as a low-grade systemic inflammatory condition, characterized by an increase in pro-inflammatory cytokines (33), and we argue that a higher rate of omega-3 PUFA intake may help reduce the risks of obesity and immune imbalance.

Increasing evidence has implicated the adaptive immune cells, such as T cells, in tempering the early innate response (e.g., 34). A number of T cell types with immunoregulatory roles have been described in the literature. Tregs contribute to the control of innate immune pathology through cytokine-dependent mechanisms and inhibition of a variety of AI and inflammatory diseases (23). CD8^+^ T cells are involved in preventing autoimmunity by releasing cytokines capable of increasing the susceptibility of target cells, or by secreting chemokines that attract other immune cells to the AI site (35). In our experiment, the populations of CD8^+^ T cell and Tregs were markedly increased in the AA rats. Therefore, we conclude that omega-3 PUFAs can exert their anti-inflammatory effect partly through T cell-mediated inhibition of the innate response.

Considering a number of results from this and other studies, we propose that in the current nutritional environment, omega-3 and -6 PUFAs exert contradictory effects on inflammation. The imbalance between the pro- and anti-inflammatory molecules in obesity, immune diseases, rheumatic diseases and cancer suggests that recovery from these diseases may benefit from an appropriate PUFA dietary therapeutic principle designed to control undesirable inflammation. Our experiments confirmed the anti-inflammatory effects of omega-3 PUFAs *in vivo*, and we ensured that models of PUFA diet-induced AA and AP rats, which are suitable for high-throughput nutrigenomic profiling experiments, were successfully established.

miRNAs regulate a variety of developmental and physiological processes, including immune homeostasis. Increasing evidence indicates that different PUFAs exert certain biological effects by direct miRNA modulation or via other metabolites (resolvins, lipoxins and protectins) that arise from PUFAs (36–39). Since miRNA deregulation caused by disproportionate ingestion of PUFAs is common, LC omega-3 PUFAs, especially DHA and EPA, may affect the expression of specific miRNAs and further protect animal tissues (40–42). However, it should be noted that most of the anti-inflammatory effects of omega-3 PUFAs have been based on *in vitro* observations. This is the first time, to the best of our knowledge, that a placebo-controlled, double blind parallel high-throughput screening strategy was adopted to elucidate the *in vivo* effects of omega-3 PUFAs on anti-inflammatory miRNAs.

Since standard rat chow already contains omega-6 PUFAs, we used the AP rats as positive controls and the Ct rats as negative controls to investigate whether additional omega-6 PUFAs may result in stronger pro-inflammatory effects, and to further illustrate the anti-inflammatory function of omega-3 PUFAs. The non-hypothesis-based high-throughput screening procedure and the high-quality AA/AP animal models employed in this study ensured the reliability of the obtained results.

Although the RT-qPCR assay is the ‘gold standard’ for verifying microarray results, it would be complex and unnecessary to attempt to validate all the differentially expressed miRNAs. Computational target prediction algorithms and functional enrichment analyses have been developed to predict the mRNA targets miRNAs and to assess the functional associations of selected gene sets (4,19,43,44). Using target prediction and enrichment analyses, we identified the MTGs that are related to immune processes and thus, successfully narrowed the range of the miRNAs to be validated by RT-qPCR (Fig. 4).

Following identification of the MTGs and prediction of their biological roles, we selected the miRNAs based on enrichment scores of the related MTG clusters in which we were interested, specifically focusing on these clusters that are involved in the regulation of immune homeostasis, and verified the expression of these miRNAs in a number of tissues. Our results showed that rno-miR-19b-3p, -146b-5p and -183-5p are significantly downregulated, and an increased level of omega-3 PUFAs can suppress inflammation *in vivo* by regulating the transcription of these three miRNAs, which in turn specifically suppress the expression of a set of inflammation-related genes. As expected, all of these genes function in vital immune signaling pathways, including the TLR, NLR, RLR, MAPK and TGF-β pathways.

Not surprisingly, computational predictions of pathway components reflected the current knowledge gained from experimental results; the predicted pathways in our study were related to the regulation of inflammation or immune homeostasis. The detection of microbes by the TLRs, NLRs and RLRs activates multiple pro-inflammatory signaling pathways to mount an effective antiviral or bactericidal response targeting the invading microbe (45–47). MAPK pathways are activated by inflammatory cytokines and environmental stresses and contribute to autoimmunity (48). TGF-β signaling generally has a negative effect on cell growth and inflammation (49). Signaling through the T-cell antigen receptor (TCR) leads to transcriptional activation of the IL-2 gene (50). An *in vitro* experiment showed that incubation of 3T3-L1 adipocytes with insulin results in a significant decrease in the miR-183-5p level (51); miR-183-5p can inhibit apoptosis in human hepatocellular carcinoma cells (52) and was found upregulated in cirrhotic liver and premalignant lesions (53). In PBMCs, miR-146b-5p may be associated with the immaturity of the neonatal immune system because it is strongly upregulated in umbilical cord blood granulocytes and T lymphocytes compared to their peripheral blood cell counterparts (54).

Although we were able to draw a schematic diagram detailing the interactions of the essential PUFA-regulated miRNAs, we still cannot determine whether the effects of omega-3 PUFAs on miRNA expression patterns are directly or are indirectly mediated by other metabolites, such as resolvin, protectin, and lipoxin, which are endogenously synthesized from PUFA precursors during inflammation (55). Moreover, because of the lack of comprehensive miRNA functional experiments and localization assays, our knowledge on the pro-resolving lipid mediators that can regulate genes involved in inflammation through the modulation of the expression of specific miRNAs remains limited (39).

Understanding how omega-3 PUFAs modulate the expression of specific miRNAs will require further investigation. In future research, with the aid of miRNA inhibition and mimicking methods, luciferase reporter systems, and *in situ* hybridization assays, we hope to explore the precise roles of the essential PUFAs in miRNA regulation and their association with pro-resolving lipid mediators. With the development of new biomedical and bioinformatic methods, comprehensive and rapid approaches for the measurement of personal miRNAomes can be established. As a consequence, specific dietary and pharmacological therapies may be developed as preventive and therapeutic strategies for autoimmune diseases.
